# Environment‐Organism Feedbacks Drive Changes in Ecological Interactions

**DOI:** 10.1111/ele.70027

**Published:** 2024-12-31

**Authors:** Oliver J. Meacock, Sara Mitri

**Affiliations:** ^1^ Department of Fundamental Microbiology University of Lausanne Lausanne Switzerland; ^2^ School of Biosciences University of Sheffield Sheffield UK

**Keywords:** antibiotic resistance, consumer‐resource modelling, context‐dependency, crossfeeding, ecological interactions, mathematical ecology, microbial communities, microfluidics, spatiotemporal dynamics, stress gradient hypothesis

## Abstract

Ecological interactions are foundational to our understanding of community composition and function. While interactions are known to change depending on the environmental context, it has generally been assumed that external environmental factors are responsible for driving these dependencies. Here, we derive a theoretical framework which instead focuses on how intrinsic environmental changes caused by the organisms themselves alter interaction values. Our central concept is the ‘instantaneous interaction’, which captures the feedback between the current environmental state and organismal growth, generating spatiotemporal context‐dependencies as organisms modify their environment over time and/or space. We use small microbial communities to illustrate how this framework can predict time‐dependencies in a toxin degradation system, and relate time‐ and spatial‐dependencies in crossfeeding communities. By re‐centring the relationship between organisms and their environment, our framework predicts the variations in interactions wherever intrinsic, organism‐driven environmental change dominates over external drivers.

## Graphical abstract

Ecological interactions are central to our understanding of the composition and function of communities, but attempts to use them as a theoretical foundation for experimental ecology have been confounded by their tendency to change depending on the environmental and spatiotemporal context. Here we develop a novel theoretical approach that shows how these context‐dependencies arise from the mechanistic basis of many interactions: the feedback between the growth of organisms and the composition of their local environment. We test our framework with small microbial communities, demonstrating the implications of environment‐organism feedbacks for the ecology of antibiotic resistance and inter‐species crossfeeding.

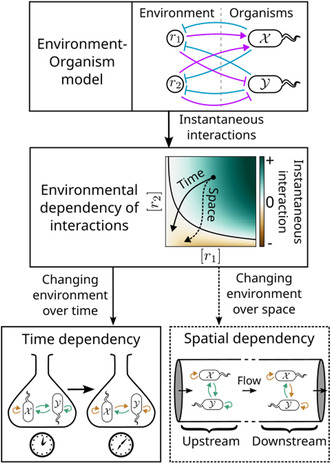

## 1 Introduction

Interactions between organisms—their impacts on each other's growth, behaviour and overall community composition (Berlow et al. 1999)—are fundamental to a bottom‐up understanding of the systems‐level properties of ecosystems. More narrowly, they are typically taken by theoretical ecologists as the per‐capita impact of one species on the net growth rate of another population (Novak et al. 2016). Though originally conceived of as fixed quantities which could be assembled into community‐level frameworks such as the generalised Lotka–Volterra (gLV) model (Volterra 1926; Lotka 1920; O'Dwyer 2018; MacArthur 1970), interactions have since been shown to often depend on the environment in which they are measured (Piccardi, Vessman, and Mitri 2019; Hoek et al. 2016; Di Martino, Picot, and Mitri 2024; Rodríguez‐Verdugo, Vulin, and Ackermann 2019; Chamberlain, Bronstein, and Rudgers 2014) and the time at which they are measured (Venkataram et al. 2023; Daniels, van Vliet, and Ackermann 2023; Chamberlain, Bronstein, and Rudgers 2014). Interactions can also be strongly influenced by spatial organisation in multi‐species communities, depending on the arrangement of partners (Nadell, Drescher, and Foster 2016; Dal Co et al. 2020). Such context‐dependencies substantially complicate bottom‐up attempts to predict community‐level outcomes based on assemblages of elementary pairwise interaction measurements (Chang et al. 2023; Friedman, Higgins, and Gore 2017). To resolve these issues, we must first understand how context‐dependencies arise and, if possible, predict them.

Numerous mechanisms can create context‐dependencies. While external environmental factors, so‐called *allogenic* factors, such as climactic change are one such component that can influence interactions (Liu and Gaines 2022; Maron, Baer, and Angert 2014), organisms can themselves influence their local environment, especially in sessile communities. Such *autogenic* environmental changes can mediate interactions when they modify the growth rate of surrounding organisms, effectively setting up a feedback loop between community composition and environmental composition. For example, reduction of local salinity by nurse plants in salt marshes can allow the establishment of salt‐sensitive species (Bertness and Shumway 1993), while environmental detoxification by partners allows toxin‐sensitive species to grow in microbial culture (Piccardi, Vessman, and Mitri 2019). In these cases, net positive interactions result as long as autogenic mechanisms that increase growth rates (such as stress buffering) outweigh mechanisms that decrease growth (such as nutrient competition). This balance shifts between different environments, resulting in a switch from net negative to net positive interactions along stress gradients (Callaway and Walker 1997; Malkinson and Tielbörger 2010; Brooker and Callaghan 1998; Di Martino, Picot, and Mitri 2024). Despite this qualitative understanding of the importance of autogenic processes in driving interaction changes, we lack corresponding theory to predict these changes and generalise the phenomena to other systems.

Here, we provide a theoretical basis for predicting environmental, temporal and spatial context‐dependencies based on the feedback between the growth rates of organisms in different environments and their autogenic impact on their environment. Our approach builds on classical consumer‐resource (CR) models, which explicitly represent the mechanisms of resource uptake through which many organisms compete (MacArthur 1970; Tilman 1980); we adopt a generalised version that incorporates inhibitory environmental factors and positive impacts of organisms on their environment (e.g., secretion of compounds), which we refer to as the environment‐organism (EO) framework (Picot et al. 2023). Prior theory relating interactions to underlying mechanisms relies on a separation of timescale approximation between environmental and species dynamics (MacArthur 1970). However, as such models cannot accurately capture the dynamics of the abiotic resources which fuel many ecosystems (O'Dwyer 2018), we adopt a novel approach that avoids this approximation. In the purely autogenic limit, we obtain an equation analogous to the gLV equation which captures environmental, temporal and spatial context‐dependencies simultaneously. Central to this expression is an interaction‐like term which depends on the environmental state, which we refer to as the ‘instantaneous interaction’. We then experimentally verify these predicted context‐dependencies using small microbial communities from which allogenic mechanisms of environmental change can be eliminated. Surprisingly, our theory reveals that apparently unconnected phenomena, such as the spatial structure of communities in microfluidic chips and the dynamics of species in batch culture, are reflections of the same underlying ecological processes. Our work thus provides a new basis for understanding the connection between interactions and their underlying mechanisms, yielding novel explanatory—and predicitive—insights.

## 2 Materials and Methods

### 2.1 Modelling

#### 2.1.1 EO Models

Our toxin‐nutrient model was derived from a pre‐existing framework (Piccardi, Vessman, and Mitri 2019), while our degrader‐crossfeeder model was built from a Monod‐based description of polymer/metabolite fluxes. We provide a complete description of these systems—including derivations of instantaneous interactions—in the Supporting Information (Sections 2.1.1–2.1.3). Details of numerical integration are provided in the Supporting Information, Section 2.1.4.

#### 2.1.2 Microfluidic Simulations

Our simulations of spatially structured flowing systems are based on a spatio‐temporal model of abundances of species and intermediates. Concentration profiles of intermediates are represented as a set of 1D scalar fields $r\left(x,t\right)$, with the position along the channel increasing from the inlet position x=0 to the outlet position x=L. Likewise, species abundances s are represented by the set of 1D scalar fields $s\left(x,t\right)$. Implicitly, we assume that the system is small enough in the y and z dimensions that it is effectively well‐mixed along these axes by diffusion, allowing us to make use of a 1D approximation.

We simulate the dynamics of the media composition using the 1D advection–diffusion equation:

(1) $$\frac{\partial r\left(x,t\right)}{\partial t}=D\frac{\partial^{2}r\left(x,t\right)}{\partial x^{2}}-v_{x}\frac{\partial r\left(x,t\right)}{\partial x}+R$$

The first term on the right represents diffusion of intermediates with diffusion coefficient D=0.5 (equal for all intermediates), chosen to ensure numerical stability of the resulting environmental trajectories. The second term represents the advective fluxes mediated by flow at a velocity $v_{x}$. We set $v_{x}$ such that advection dominates over diffusion given the channel length L and the diffusion coefficient D (i.e., that the Péclet number $\mathrm{Pe}=\frac{{\mathrm{Lv}}_{x}}{D}$ is substantially greater than 1), a necessary condition of our framework (Supporting Information Text S4). The final term represents the sources and sinks of intermediates at each position, in this case given by an adjusted form of the impact functions for the degrader‐crossfeeder model (Equation S23). Together, these terms give the total rate of change in the intermediate concentrations at a point x in the channel. Microbial population dynamics are simulated at each spatial location and are assumed to be unaffected directly by diffusion or flow, with local growth rates based on an adjusted form of the sensitivity functions (Equation S22). In brief, these adjustments consist of the addition of a chemostat‐like mortality term $\theta =0.005v_{x}$ which accounts for wash‐out of microbes by flow and a maximal channel occupancy λ. $v_{x}$ was selected as 10 in Figure 4 to obtain a balance between refreshment of media and excess washout of cells, similar to the experimental procedure described in Daniels, van Vliet, and Ackermann (2023). The results of other values of $v_{x}$ are shown in Figure S8. Further details are provided in the Supporting Information, Section 2.1.5.

### 2.2 Experiments

#### 2.2.1 Strains and Growth Conditions

Our *Comamonas testosteroni* strain MWF001 is described in Piccardi, Vessman, and Mitri (2019). Cells were streaked onto TSA plates from freezer stocks and grown overnight. Single colonies were then picked (one colony per biological replicate), and cells were grown overnight in glass Erlenmeyer flasks under continuous shaking in base minimal media (Table S1) supplemented with 10 mM proline. Due to the slow growth of *C. testosteroni* under these conditions, cells were in exponential phase at the end of this period. Cells were then washed twice in PBS. The OD
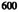
s of the washed cultures were then measured and cultures diluted to initialise experiments at the appropriate starting densities as described below. Cultures were grown at 28°C in all cases.

#### 2.2.2 Intraspecific Interaction Measurements

We prepared 96‐well plates with 180 μL of basal media supplemented with varying concentrations of proline ([pro]_0_ = 0.5, 1, 2, 5 mM) and ampicillin ([amp]_0_ = 0, 10, 20, 30 μg mL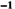). Twenty microlitres of an exponential‐phase culture of *C. testosteroni* was then added, with three wells of each condition containing culture adjusted to high density (OD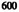 = 0.004) and three wells containing culture adjusted to low density (OD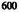 = 0.001). The plate was placed into a plate reader (BioTek Synergy H1) and OD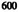 readings for each well were taken every 30 min for 120 h at 28°C under continual shaking between timepoints.

The background signal was subtracted from the resulting raw growth curves by first estimating the initial OD contribution from cells in the high inoculation OD wells (κ) using the equation

(2) $$\kappa =\frac{4}{3}\left(<{\mathrm{OD}}_{h}\left(0\right)>-<{\mathrm{OD}}_{l}\left(0\right)>\right)$$

where $<{\mathrm{OD}}_{h}\left(0\right)>$ and $<{\mathrm{OD}}_{l}\left(0\right)>$ represent the plate‐wide average initial OD readings for the high inoculation density and low inoculation density wells respectively. The factor of $\frac{4}{3}$ stems from the 1:4 inoculation density ratio. Each curve was individually adjusted by subtracting the average OD of the specified curve's first three timepoints and adding either κ for the high inoculation density wells or $\frac{\kappa }{4}$ for the low inoculation density wells. The average OD curves were then calculated from the three replicates for each condition and used to calculate the measured interactions as described in the main text.

## 3 Results

### 3.1 A Theoretical EO Interaction Framework Explains Multiple Context‐Dependencies

EO systems can be modelled by breaking them into three parts (Meszéna et al. 2006; Tilman 1980; Koffel, Daufresne, and Klausmeier 2021): firstly the *impact function* of a species β, $f_{\beta }\left(r\right)$ describes the rate at which one unit of β modifies its environment, that is, the autogenic component of environmental change. We denote this environment with the vector r, which we will mostly take here to represent the concentrations of different chemical intermediates (e.g., element 1 represents the concentration of glucose, element 2 acetate), but may more generally represent quantities such as temperature and light availability. r defines a position in the ‘environment space’, the set of different possible environmental states. The impact function is itself dependent upon r, allowing it to capture, for example, concentration‐dependent uptake of a resource. r is also affected by the second EO component $\sigma \left(r\right)$, which represents allogenic processes such as flows of intermediates into or out of the system. We can then write the rate of change in the environment as:

(3) $$\frac{dr}{\mathrm{dt}}=\sum_{\beta }s_{\beta }f_{\beta }\left(r\right)+\sigma \left(r\right)$$

where $s_{\beta }$ is the instantaneous abundance of species β.

Thirdly, the *sensitivity function* $g_{\alpha }$ describes the per‐capita growth rate of a species α in a particular environment:

(4) $$\frac{1}{s_{\alpha }}\frac{{\mathrm{ds}}_{\alpha }}{\mathrm{dt}}=g_{\alpha }\left(r\right).$$

As defined here, these functions are very general, allowing the expression of various categories of EO relationship. These include ‘switching’ phenotypes such as diauxy, as well as combinations of essential resources (Tilman 1980). Equations (3) and (4) are essentially identical to typical CR formulations (Cui, Marsland, and Mehta 2024), though generalised to allow elements of $f_{\beta }\left(r\right)$ to be positive (representing secretion) and to allow $g_{\alpha }\left(r\right)$ to be negatively influenced by components of r (representing toxicity).

**FIGURE 1:**
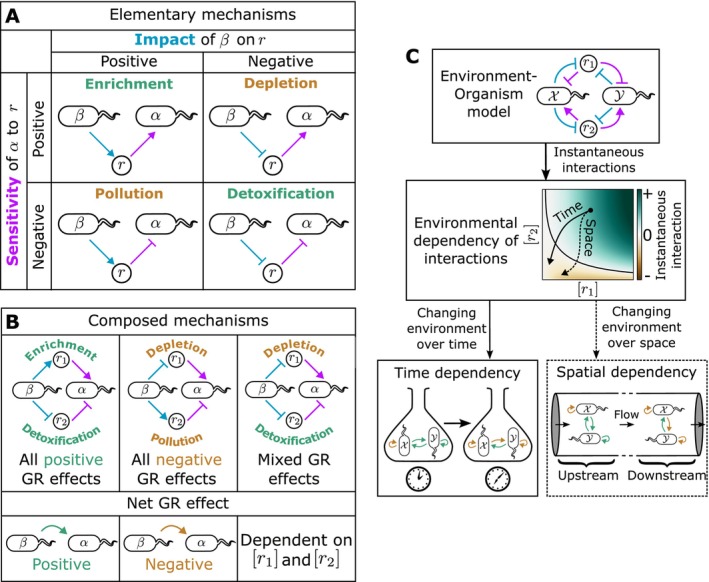
Multiple interaction context‐dependencies can be explained with a single theoretical framework. (A) Environmentally mediated interactions between organisms can be broken into elementary components by considering the role of each environmental factor $r_{\rho }$ separately. ‘Sensitivity functions’ (purple) denote the effect of increasing a factor r on the growth rate of a target species α (i.e., whether it decreases—bar—or increases—arrow—α's growth), while ‘impact functions' (blue) denote the effect of an effector species β on r (i.e., whether it is increased—arrow—or decreased—bar). Combinations of these functions imply four elementary interaction mechanisms: Enrichment and detoxification which enhance the growth of α, and depletion and pollution which reduce α's growth (Estrela et al. 2019; Koffel, Daufresne, and Klausmeier 2021). (B) Elementary mechanisms can be composed together through different environmental factors, with the net growth rate (GR) impact depending on the combined effect of the composed elements. When elementary mechanisms with both positive and negative effects are mixed, the sign of the net effect depends on the balance between the factors—that is, the environmental context. (C) Our framework shows how EO models give rise to an instantaneous interaction that depends on the environment. As the environment changes over time (e.g., in batch culture) or over space (e.g., in microfluidic channels at steady‐state), this environmental‐dependency in turn gives rise to time and spatial dependencies.

The dependence of $g_{\alpha }$ on r implies that environmental changes caused by both α itself (β=α, intraspecific interactions) and other species (β≠α, interspecific interactions) (Equation 3) will regulate α's growth rate. Breaking this regulation into the effect mediated by each environmental factor $r_{\rho }$ individually, we can define four types of elementary mechanisms categorised by the combinations of the signs of the impact and sensitivity functions. Following recently defined terminology for metabolic interactions (Koffel, Daufresne, and Klausmeier 2021; Estrela et al. 2019), we refer to these elementary mechanisms as enrichment (β produces a nutrient that enhances the growth of α), depletion (β reduces the concentration of a nutrient, impeding the growth of α), pollution (β produces a toxin that impedes the growth of α) and detoxification (β decreases the concentration of a toxin of α and enhances its growth) (Figure 1A).

Species can interact through multiple environmental factors $r_{\rho }$. The net impact on growth then results from the summation of the effects of each of the composed elementary mechanisms at play. Most interesting are cases where the composed mechanisms have a mixture of positive and negative impacts (e.g., depletion combined with detoxification), in which case the net effect will depend on the relative balance of the positive and negative mechanisms, in turn dependent upon the environmental context (Figure 1B). This environmental‐dependence arises naturally within the EO framework: in systems dominated by autogenic mechanisms of environmental change—such that σ=0—it can be shown that (Supporting Information Text S1):

(5) $$\frac{1}{s_{\alpha }}\frac{{\mathrm{ds}}_{\alpha }}{\mathrm{dt}}=g_{\alpha }\left(r_{0}\right)+\sum_{\beta }\int_{0}^{t}a_{\alpha \beta }^{\prime }\left(r\right)\;s_{\beta }\;\mathrm{d\tau}$$

where $r_{0}$ is the initial environmental composition and the integral is taken over the entire history of the system up to the current time t (parameterised by τ). This expression is derived by considering a path integral through the environment space and precisely disentangles the impact of each species on the growth rate of all other species through autogenic environmental change. We will refer to it as the closed environment‐organism (cEO) equation, as the environment is closed with respect to allogenic influences.

Central to this expression is the term $a_{\alpha \beta }^{\prime }\left(r\right)$, which we call the *instantaneous interaction* by analogy to the gLV equation:

(6) $$\frac{1}{s_{\alpha }}\frac{{\mathrm{ds}}_{\alpha }}{\mathrm{dt}}=\mu_{\alpha }+\sum_{\beta }a_{\alpha \beta }\;s_{\beta }$$

Here, $\mu_{\alpha }$ is α's intrinsic growth rate (i.e., its growth in the absence of other species and at low population sizes) and $a_{\alpha \beta }$ is the interaction between β and α (i.e., the density‐dependent impact of β on the growth rate of α).

While analogous in structure, we note some important differences between the cEO and gLV equations: firstly, in contrast to the fixed gLV interactions $a_{\alpha \beta }$, $a_{\alpha \beta }^{\prime }\left(r\right)$ is dependent on the environment r. This arises from its definition as the composition of the environmentally dependent impact and sensitivity functions:

(7) $$a_{\alpha \beta }^{\prime }\left(r\right)\equiv \nabla g_{\alpha }\left(r\right)\cdot f_{\beta }\left(r\right)$$

$\nabla g_{\alpha }\left(r\right)$ is the gradient of the sensitivity function, a vector field which denotes the direction in the environment space along which the growth rate of α increases most rapidly. The scalar product of this with $f_{\beta }\left(r\right)$ (also a vector field) therefore indicates whether β is pulling the environment in a direction that increases (positive $a_{\alpha \beta }^{\prime }$) or decreases (negative $a_{\alpha \beta }^{\prime }$) the growth rate of α at a given r. Second, this environmental dependence is not static—in contrast to equilibrium gLV systems, environments under autogenic control are generally out of equilibrium and trace out a trajectory $r\left(t\right)$ in the environment space, over which $a_{\alpha \beta }^{\prime }\left(r\right)$ can vary substantially. In Supporting Information Text S1, we additionally show that $a_{\alpha \beta }^{\prime }$ arises in equilibrium systems that can be described by the gLV equation (such as chemostats) where it plays an equivalent role to the interaction matrix (Novak et al. 2016). We can exploit this link to estimate interactions in equilibrium settings by isolating the environment from allogenic influences and measuring the resulting nonequilibrium growth rate dynamics of pairs of organisms (Supporting Information Text S2).

Finally, growth rate impacts in the EO framework are cumulative, arising from the integration of the instantaneous interaction term over the entire history of the system up to the current time t ($\int_{0}^{t}a_{\alpha \beta }^{\prime }\left(r\right)\;s_{\beta }\;\mathrm{d\tau}$). This is because interactions are mediated via ongoing changes to environmental factors, which take time to be impacted by organisms. We refer to the resulting net impact of β on α's growth rate—considering all autogenic environmental changes caused by β up to this point—as the *cumulative interaction*.

In the remainder of this manuscript, we illustrate how the environmental‐dependency of the instantaneous interaction results in time‐dependencies and spatial structure that can be predicted if the underlying elementary mechanisms (represented by appropriate choices of the impact and sensitivity functions) are known (Figure 1C).

### 3.2 Mixed Mechanisms Can Result in Interaction Time‐Dependencies

**FIGURE 2:**
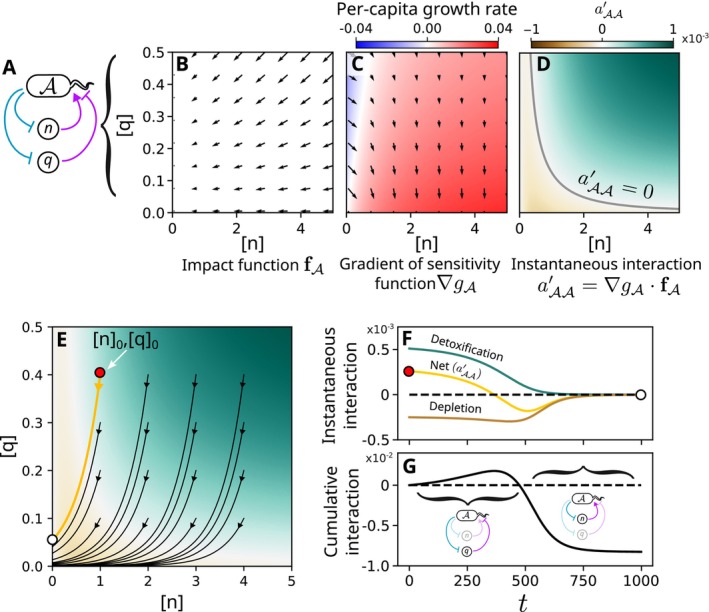
Intraspecific interactions mediated by mixtures of positive and negative mechanisms are predicted to switch sign over time in batch culture. (A) One of the simplest examples of a system with mixed elementary mechanisms is a single species A which increases the growth of other members of its population by detoxifying an environmental toxin while reducing their growth by depleting a common nutrient. (B, C) We can represent the impact and sensitivity functions for A using the ‘environment space’, which denotes the values of the different growth‐limiting environmental factors (in this case, the concentrations of the nutrient $\left[n\right]$ and of the toxin $\left[q\right]$). Impact functions are vector fields sitting in this space (black arrows, B), while sensitivity functions are scalar fields (C). The gradient of the sensitivity function then represents the direction in the environment space in which the growth rate of A increases most rapidly, as well as how quickly it increases (black arrows, C). (D) Taking the scalar product of the impact function and the gradient of the sensitivity function yields the instantaneous interaction $a_{\mathrm{AA}}^{\prime }$, representing the instantaneous effect that A has on its own growth rate at a given position in the environment space. (E) Purely autogenic systems such as batch culture experiments trace out trajectories in this environment space, starting from an initial position ${\left[n\right]}_{0}$, ${\left[q\right]}_{0}$. (F) Considering a single trajectory with ${\left[n\right]}_{0}=1$, ${\left[q\right]}_{0}=0.4$, we can calculate both the net instantaneous interaction $a_{\mathrm{AA}}^{\prime }$ and the contributions from the two elementary mechanisms as a function of time. (G) The integrated effect of A on its own growth (the cumulative interaction) demonstrates a switch in the intraspecific interaction: At early timepoints, when the toxin concentration is high, detoxification dominates and the interaction appears positive. By contrast, at late timepoints when the toxin has mostly been removed, depletion of the single nutrient dominates and the interaction becomes negative.

One of the simplest mixtures of mechanisms with opposing effects consists of a single species A interacting negatively with itself via nutrient depletion and positively via detoxification (Figure 2A). We modelled this system using Monod‐based impact (Figure 2B) and sensitivity functions (Figure 2C) describing the dynamics of the toxin, nutrient and cell abundances (Supporting Information). Placing this system in a closed batch culture setting prevents allogenic influxes of intermediates and thus satisfies the purely autogenic assumption of the cEO equation. The instantaneous intraspecific interaction $a_{\mathrm{AA}}^{\prime }$ (Figure 2D) recapitulates the environmental‐dependency of interactions in this system, with positive intraspecific interactions dominating at high toxin concentrations and negative intraspecific interactions at low toxin concentrations (Piccardi, Vessman, and Mitri 2019). The environment space is traversed by the system as it evolves from some initial state $r_{0}$, following the trajectory $r\left(t\right)$. In this case, the system moves towards the origin as A reduces the concentration of both the nutrient $\left[n\right]$ and the toxin $\left[q\right]$, with the exact trajectory varying depending on the initial environmental context (Figure 2E). This means that the instantaneous interaction $a_{\mathrm{AA}}^{\prime }$ can switch signs from positive to negative over time because it captures the net effect, where the dominance of the two mechanisms switches from detoxification to depletion (Figure 2F).

This switch in sign of the instantaneous intraspecific interaction propagates through to A's growth rate. As there are no other species in this system, the sole growth rate effect is the time‐dependent impact of A on itself—the cumulative intraspecific interaction—given by $\int_{0}^{t}a_{\mathrm{AA}}^{\prime }\left(r\right)\;s_{A}\;\mathrm{d\tau}$. This switches from positive to negative once the accumulated benefit of the removal of the toxin is outweighed by the accumulated penalty from the reduction in the nutrient concentration (Figure 2G). We therefore predicted from this model that measurements of the intraspecific interaction in such systems should give positive values if performed early on (when detoxification dominates) and negative values if performed later (when depletion dominates).

### 3.3 An Antibiotic‐Based Experimental System Demonstrates Sign‐Switching of the Intraspecific Interaction

**FIGURE 3:**
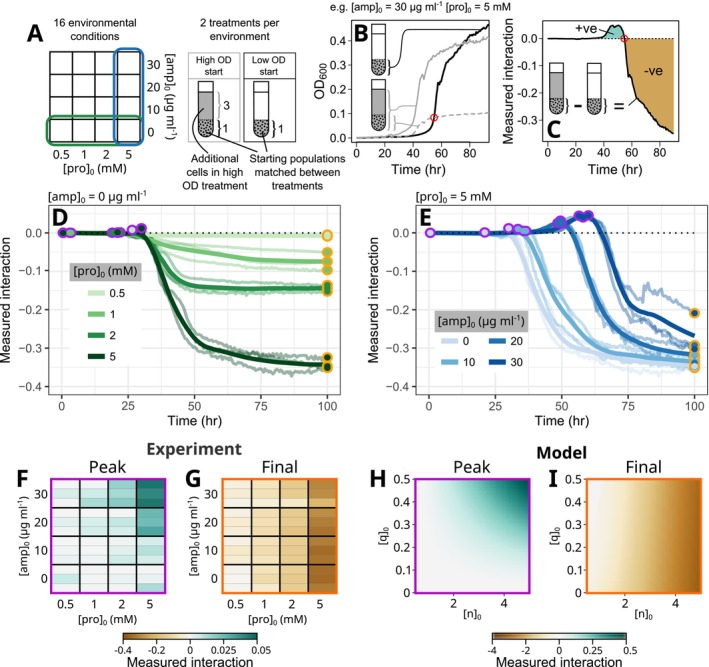
An antibiotic‐based model system demonstrates sign switching of measured intraspecific interactions over time. *Comamonas testosteroni* is a β‐lactamase producing soil bacterium which can degrade environmental ampicillin. Combined with competition over a single limiting carbon source (proline), we used this as an experimental analogue of the model shown in Figure 2. (A) Exponential‐phase cells were transferred to a 96‐well plate containing wells with different initial ampicillin concentrations [amp]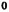 and proline concentrations [pro]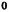. Six wells were prepared for each condition, consisting of three replicates each of low and high initial inoculation densities at a 1:4 density ratio, allowing 16 different environmental conditions in total. (B, C) We measured the growth curve of each well and averaged the technical replicates. We then calculated the measured interaction over time by normalising the averaged high optical density (OD) curve by the ratio of the starting ODs (B) and subtracting the low OD curve (main text, Figure S2, Supporting Information Text S3) (C). Measured interactions greater than 0 indicate that growth of a matched subpopulation of *C. testosteroni* (black dots) was enhanced by the presence of additional members of the same species in the high‐OD wells relative to the low‐OD wells (a positive intraspecific interaction), while differences less than 0 indicate growth suppression (a negative intraspecific interaction). (D, E) Comparing measured interactions across different proline (D) and ampicillin (E) concentrations demonstrates the environment‐dependent shift in positive to negative interactions predicted by the model. We summarise this shift for each condition by measuring the peak (purple circles) and final (orange circles) measured interactions for each condition (F, G). These qualitatively match predictions from our modelling framework (H, I). The general pattern that emerges from these simulations is robust to changes in simulation parameters (Figure S4). Faint lines in (D and E) indicate n=3 separate biological replicates performed on separate days, while bold lines indicate LOESS‐smoothed averages. Biological replicates are indicated in (F) and (G) by separate horizontal strips.

We now investigated whether this prediction was borne out experimentally. We used a bacterium ( *Comamonas testosteroni* ) that can degrade β‐lactam antibiotics via induced secretion of β‐lactamases (Figure S1) and which competes with itself over proline as a sole carbon source as an experimental analogue of the detoxification/depletion system shown in Figure 2A. To measure intraspecific interaction changes over time in multiple environmental contexts, we prepared arrays of environmental conditions (with varying initial proline, [pro]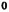 and ampicillin, [amp]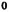, concentrations) within 96‐well plates (Figure 3A). Each condition was split into two sets of wells, one inoculated with exponential‐phase *C. testosteroni* cells at high density and the second at low density. Absorbance‐based growth curves of these cultures were then measured in a plate reader, which was used to calculate a quantity we call the *measured interaction* (for a comparison of the three interaction concepts we discuss in this paper—instantaneous, cumulative and measured—please refer to Supporting Information Text S3 and Figure S2).

Analogous to existing experimental measurements of interactions, in which the growth of a focal population is measured in the presence or absence of a partner (Piccardi, Vessman, and Mitri 2019; Mitri and Foster 2013; Foster and Bell 2012; Kehe et al. 2021), we can treat our low inoculation density condition as a ‘monoculture‐like’ assay, with a corresponding subpopulation in the high‐density condition which is of equal size. In the high‐density condition, this subpopulation is effectively cocultured with a second subpopulation of the same species. We can therefore measure the intraspecific interaction by comparing the fate of the matching subpopulations in the high‐ and low‐density conditions (Figure 3B). This is achieved by dividing the growth curve of the high‐density culture by the ratio of inoculation densities (4:1), yielding the size of the subpopulation as a function of time. At times when this normalised curve is higher (lower) than that of the low inoculation density condition, we can infer that the presence of additional cells of the same species enhanced (reduced) the subpopulation's growth—that is, that a positive (negative) intraspecific interaction has occurred (Figure 3B). We can therefore simply subtract the low inoculation density curve from the normalised high inoculation density curve to infer the interaction (Figure 3C). We considered several alternative definitions of the measured interaction (Figure S2D–G), but found that this abundance difference provided the optimal balance between capturing the shape of the cumulative interaction and robustness to measurement noise. We note that it represents a time‐varying version of accepted endpoint‐based interaction metrics (Foster and Bell 2012).

Beginning with the control conditions with zero antibiotic, the low inoculation density curves looked similar to the high inoculation density curves aside from a consistent lag (Figure S3A). The ratio of densities between the two conditions remained approximately equal to the inoculation ratio until the high‐density condition approached stationary phase, implying this lag arises from the smaller initial number of cells in the low‐density condition. This is reflected in the measured intraspecific interaction, which was approximately neutral up to this point and negative afterwards (Figure 3D). In the presence of antibiotics, the lag between the two conditions increased, presumably because the smaller initial population was slower to degrade the ampicillin before starting to grow (Figure S3B). Consequently, we observed a concentration‐dependent positive interaction emerging with increasing [amp]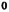, as predicted by the model (Figure 3E). Ultimately, all environments resulted in negative interactions in the long term. Summarising these time‐dependencies by considering the peak and final measured interactions demonstrates the environmental and time‐dependencies together (Figure 3F,G), which qualitatively match the predictions of our modelling framework (Figure 3H,I). Although we do not directly fit model parameters to our data, we find that these qualitative patterns are robust to large changes in parameter values, suggesting that these results are not a result of fine‐tuning of the model (Figure S4).

Evolutionary rescue can result in similar abundance trajectories as those described here, as a small number of mutant cells with antibiotic‐resistant genotypes can grow to fixation after a long lag (Orr and Unckless 2014; Ramsayer, Kaltz, and Hochberg 2013). We tested whether evolution could play a role in our experimental system by measuring the MIC of ampicillin for each culture at the end of our interaction measurement timecourses (Figure S5). While we did observe a small increase (≈ 50%) in the resistance of populations exposed to the highest ampicillin concentrations compared to those grown under antibiotic‐free conditions, simulations incorporating the evolution of resistance showed that evolutionary trends, far from driving the observed interaction time‐dependencies, tend to attenuate measured positive interactions if they have any effect at all (Figure S6). Thus, we concluded that the consistent positive‐to‐negative interaction switch that we observe arises from the changing dominance of the two elementary interaction mechanisms, as suggested by our theoretical framework.

### 3.4 Small Crossfeeding Communities Illustrate the Common Origins of Time‐Dependent Interactions and Spatial Structure

**FIGURE 4:**
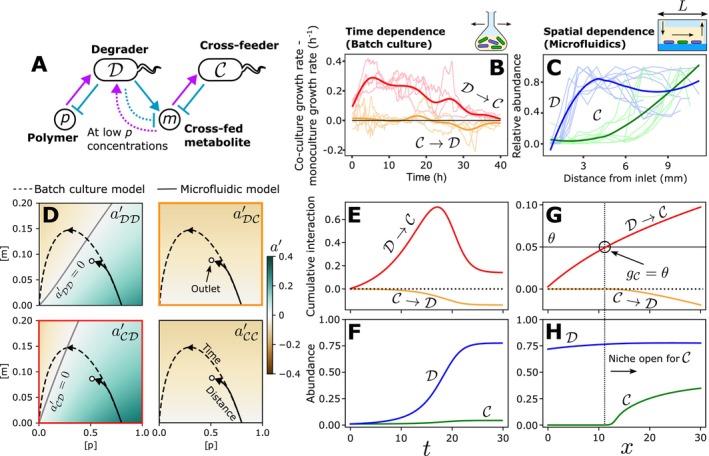
Our framework shows that interaction time‐dependencies and spatial structure can arise from closely related processes. (A) Two recent studies (Daniels, van Vliet, and Ackermann 2023; Wong et al. 2023) describe the ecological patterns arising in a two‐species community consisting of a degrader D that consumes a polymer p and produces a metabolic by‐product m which is consumed by a second crossfeeding species C. At low concentrations of p, D switches from net production of m to consumption. (B) Daniels, van Vliet, and Ackermann (2023) find that this type of community displays time‐dependent interspecific interactions in batch culture, with the impact of D on C increasing early on and decreasing later (red) and the impact of C on D switching from neutral to negative (orange). (C) By contrast, Wong et al. (2023) show how a similar community patterns itself in microfluidic channels of length L with unidirectional flow, with C only being able to grow towards the outlet of the device. (D) We constructed an EO model of this community and applied our analytical techniques to obtain the instantaneous interaction matrix for each possible pair of community members (main text, Supporting Information). We then simulated the environmental trajectories of batch culture (dashed lines) and the microfluidic device (solid lines) inoculated with this community (Methods). In the case of the microfluidic device, the initial environment ${\left[p\right]}_{0},{\left[m\right]}_{0}$ corresponds to the composition of the media injected into the system at the inlet, while points along the environmental trajectory indicate the steady‐state media composition at different positions along the channel. (E, F) In the batch‐culture model, the gradual enhancement of the environment by D for C via conversion of p to m results in a gradual increase in the cumulative interaction from D to C. Later, once p has been largely exhausted, the switch in the behaviour of D from net production to net uptake of m leads to competition between the two species, and a downward trend in both interspecific cumulative interactions (E). These dynamics are difficult to dissect from the raw growth curves (F). (G, H) When this community is placed into the spatial context of a simulated microfluidic channel with flowrate $v_{x}=10$, we observe a similar interaction pattern from the inlet to the outlet, with a positive interaction accumulating from D to C. At a certain position, this positive cumulative interaction exceeds the mortality rate θ representing the flushing of cells by flow. Beyond this point, the net growth rate of C is positive, reflecting the opening of a niche for C (G). This leads to the spatial structuring of the two species observed in experiments (H).

So far, we have considered time‐ and environmental‐dependencies in a mono‐species system. However, our framework generalises to multi‐species communities, as well as certain types of spatially structured communities (Methods, Supporting Information Text S4, Figure S7). Two recent studies have described time‐ (Daniels, van Vliet, and Ackermann 2023) and spatial‐ (Wong et al. 2023) dependencies of similar two‐species communities. In both cases, a degrader species D consumes a polymer (chitin or dextran) and subsequently produces a metabolite (acetate or glucose) which is consumed by the second crossfeeding community member C. When the polymer is exhausted, D can switch from net production to net consumption of the crossfed metabolite (Figure 4A). Daniels, van Vliet, and Ackermann (2023) observed a time‐dependency of the interspecific interactions in batch culture (Figure 4B), while loading of a similar community into a microfluidic channel under flow resulted in spontaneous self‐structuring of the community along the channel with C only growing towards the outlet (Wong et al. 2023; Figure 4C). Given the commonalities between the two studies, we decided to use them as case studies for how our framework can unify similar observations occurring across time or space.

We built an EO model of such degrader/crossfeeder communities and derived expressions for the four different instantaneous interactions $a_{\mathrm{DD}}^{\prime }$, $a_{\mathrm{DC}}^{\prime }$, $a_{\mathrm{CD}}^{\prime }$ and $a_{\mathrm{CC}}^{\prime }$ (Supporting Information). As shown in Figure 4D, these four quantities can be arranged analogously to the interaction matrix of the gLV framework, with intraspecific interactions located along the main diagonal and interspecific interactions located off this axis. However, instead of being represented by a single value as in the gLV model, the instantaneous interactions are expanded into scalar fields defined on the entire environment space, capturing the environmental‐dependency of each interaction. Both the degrader's intraspecific instantaneous interaction $a_{\mathrm{DD}}^{\prime }$ and interspecific instantaneous interaction $a_{\mathrm{CD}}^{\prime }$ contain positive and negative regions, reflecting the changing balance between the enrichment mechanism (production of the crossfed metabolite from the polymer) and the depletion mechanism (competition over the crossfed metabolite) in different environments.

In batch culture, organisms modify their environment by secreting and consuming intermediates over time. A similar effect occurs in flowing systems, whereby the intermediates within a parcel of fluid are sequentially modified by the organisms residing at successive spatial locations as it is transported downstream. This results in an environmental trajectory in space that is formally equivalent to the temporal trajectory of batch culture systems when the spatial system is allowed to reach a steady state (Supporting Information Text S4). This allows us to directly compare the batch culture system trajectory (Figure 4D, dashed lines) and the microfluidic trajectory (Figure 4D, solid lines) when plotted onto the instantaneous interaction maps, enabling us to interpret the changing interactions over time and space using the same framework. Both systems sweep out initial paths with similar shapes, suggesting that the temporal patterning of the batch culture and the spatial patterning of the channel may arise from similar changes in interaction strengths.

To explore this in more detail, we now broke down the growth dynamics in the batch culture simulations into cumulative interactions, focusing on the interspecific cases (Figure 4E,F). We observed a similar pattern of time evolution in the batch culture interactions as in the original study (Figure 4B,E). In the initial phase, the large initial amount of polymer is metabolised by the degrader, resulting in large amounts of free metabolite. While this substantially enhances the growth of the crossfeeder, the low utility of the metabolite at this point for the degrader prevents the crossfeeder from having a strongly negative impact on the degrader. Later, the switch of the degrader to net metabolite uptake leads to mutual competition between the two species, decreasing the strength of the net‐positive interaction with the crossfeeder and causing a net‐negative impact of the crossfeeder on the growth of degrader.

Similar effects arise in the spatially structured system (Figure 4G,H). The crossfeeder cannot grow near the inlet as the rate at which it is washed out of the device (θ) exceeds the growth rate sustained at very low metabolite concentrations. However, the activity of the degrader leads to a gradual enhancement of the environment for the crossfeeder along the length of the channel and ultimately leads to the opening of a new niche when the cumulative interaction from the degrader to the crossfeeder exceeds the threshold set by θ. This generates spatial structure, with the crossfeeder only growing towards the outlet of the device (Figure 4C,H). Our model also reproduces the suppressive effect of increased flow rates on the growth of C, as observed experimentally (Wong et al. 2023) (Figure S8). In summary, our framework shows how spatial patterns arising under uni‐directional flow and interaction time‐dependencies in well‐mixed systems are reflections of the same underlying ecological processes.

## 4 Discussion

We have presented a general framework that explains context‐dependencies of interactions as arising from feedback between organisms and their environment. This viewpoint provides a theoretical justification for the ubiquity of context‐dependencies of environmentally mediated interactions (Chamberlain, Bronstein, and Rudgers 2014; He, Bertness, and Altieri 2013; Shantz, Lemoine, and Burkepile 2016): aside from some carefully chosen combinations of the impact and sensitivity functions, Equation (7) implies that essentially every environmentally mediated interaction will depend on the environmental state. Furthermore, as organisms often change their environment over time, interaction changes over time should be widespread. Our single‐species toxin/nutrient system (Figures 2, 3) provides an illustration of this effect. Initially, the population interacts positively with itself (increases its own growth rate) through environmental detoxification, but this mechanism inherently causes a sign switch of the interaction: once the toxin is eliminated, the positive interaction mechanism is suppressed and competition for the nutrient dominates. The autogenic environmental changes thus effectively set up a stress gradient in time, driving the observed time‐dependency of the interaction (Brooker and Callaghan 1998).

Our results have particular relevance for our understanding of the outcomes of batch culture interaction measurements (Piccardi, Vessman, and Mitri 2019; Mitri and Foster 2013; Foster and Bell 2012; Kehe et al. 2021; Hsu et al. 2019; Weiss et al. 2021). The mechanism by which measured interactions in batch culture switch from positive to negative once nutrients become limiting (Figure 3) is quite general and suggests that measurements based on end‐point abundances may miss positive interactions during early community establishment. This may at least partially explain the ongoing controversy surrounding the relative distribution of negative and positive interactions in natural communities (Palmer and Foster 2022; Yu et al. 2022; Kehe et al. 2021; Zelezniak et al. 2015; Foster and Bell 2012).

More broadly, we see two general applications of this work. First, we show that placement of the same community of organisms in different types of system can result in distinct but connected phenomena. For example, in Figure 4 we show how time‐dependencies in batch culture and spatial structure in flowcells are manifestations of the same EO feedbacks. Of more practical relevance, we discuss in Supporting Information Texts S1 and S2 a novel route by which our framework allows one to map interactions in open, equilibrium systems like chemostats through measurements of closed experimental systems like batch cultures, thereby providing a novel basis for evaluating the ecology of open ecosystems. Crucially, this approach is based purely on species abundance measurements, meaning it can be applied even when the underlying environmental dynamics are unknown. This insight might be used to explore the ecological landscape around equilibrium states in chemostat‐like systems by applying environmental perturbations in batch culture, potentially opening up new routes to rationally control community composition by changing the environment (Goyal, Rocks, and Mehta 2024; Sánchez et al. 2024).

Secondly, our work provides new theoretical tools for connecting the underlying mechanisms of species' interactions to their evolution and ecology. The instantaneous interaction $a_{\alpha \beta }^{\prime }$ we discuss throughout this work is also at the heart of the ecology of the equilibrium systems typically of interest to theoreticians (Supporting Information Text S1, (Koffel, Daufresne, and Klausmeier 2021)). As it can be calculated for most realistic choices of the impact and sensitivity functions (Equation 7), it represents a much more flexible link between mechanistic models and interaction frameworks than the separation of timescales approach (MacArthur 1970; O'Dwyer 2018). We anticipate that this link will allow deeper mechanistic insights into phenomena such as higher order interactions (HOIs) (Sanchez 2019; Abrams 1983; Billick and Case 1994; Gibbs, Levin, and Levine 2022) and the evolution of environmentally mediated social traits (Govaert et al. 2019), as well as the dependence of such *evolutionary* outcomes on the environmental context (Drew, Stevens, and King 2021).

We also note that despite our focus on microbial ecosystems, our results should also hold true for macroscopic ecosystems as long as the assumptions of our framework—particularly our assumption of autogenic dominance—are at least approximately true. Indeed, the interplay between organisms and their environment has long been understood to drive primary succession in plant ecosystems, whereby modification of the local environment by early pioneer species opens new niches and eventual replacement of pioneers by latecomers better adapted for the new environment (Roberts 1987; Connell and Slatyer 1977). In Supporting Information Text S5 and Figure S9, we illustrate this idea with a model of autogenic primary succession of plants. Calculation of the cumulative interactions illustrates the complex time‐dependency of the interactions in this system, with some changing signs (Figure S9E). Similar successional patterns are observed in macroscopic systems such as whale falls (Smith et al. 2015) and microscopic systems such as marine snow (Pontrelli et al. 2022; Datta et al. 2016) in which allogenic nutrient fluxes are substantially smaller than the autogenic impacts of detritivores. Likewise, the spatial niche‐opening effects we describe in flowing systems may at least partially explain the longitudinal patterning of organisms in systems such as rivers and the gut (Vannote et al. 1980; Riva et al. 2019; Pereira and Berry 2017).

Nevertheless, there are some limitations to our framework. While we can generalise our framework to incorporate allogenic mechanisms (Equation S4), in most of this work we have assumed that allogenic factors can be eliminated. This assumption allows the cEO and gLV equations to be analogised, but is not applicable to the numerous communities which are subject to external influences. Nevertheless, this assumption is less limiting than it may initially appear, covering for example nutrient cycling in closed environments (de Jesús Astacio et al. 2021). We also do not specify how the initial environment $r_{0}$ is reached. This is an external constraint which must be carefully selected, for example, based on the composition of virgin substrate in the case of a primary succession. Another important choice—particularly when attempting to directly compare models and experiments—is the selection of the impact and sensitivity functions. For large ecosystems, the number of environmental factors involved and the difficulty in measuring them can make accurate determination of these functions challenging. However, recent work suggests they can be constrained by *in silico* approaches such as genome‐scale modelling (Schäfer et al. 2023) without extensive experimental investigation. Lastly, our assumption that interactions are environmentally mediated, while well‐grounded for many microbial and plant communities (Gralka et al. 2020; Roberts 1987), cannot account for direct interaction mechanisms such as predation and contact‐dependent processes (Sockett 2009; Hayes, Aoki, and Low 2010).

In summary, our work shows that many context‐dependencies of ecological interactions can be explained by reciprocal feedback between the growth of organisms and their resulting environmental impacts. Knowledge of these feedbacks can be used to predict and interpret interaction changes, providing a path forward in the effort to manipulate interactions to predictable ends. Ultimately, we anticipate that a renewed focus on the role of the environment in dynamical ecosystems will open new methods for controlling communities, as well as help to resolve longstanding questions regarding their composition and diversity.

## Author Contributions

O.J.M. and S.M. conceived the study and edited the manuscript. O.J.M. developed models, performed experiments, analysed data and wrote the manuscript.

### Peer Review

The peer review history for this article is available at https://www.webofscience.com/api/gateway/wos/peer‐review/10.1111/ele.70027.

## Supporting information


Data S1.

## Data Availability

All data and code used in this study (apart from data reproduced from other studies: Figure 4B,C) are available at https://doi.org/10.5281/zenodo.13018090.
